# Permeabilization of the mycobacterial envelope for protein cytolocalization studies by immunofluorescence microscopy

**DOI:** 10.1186/1471-2180-6-35

**Published:** 2006-04-18

**Authors:** Mena Cimino, Lorenzo Alamo, Leiria Salazar

**Affiliations:** 1 Instituto Venezolano de Investigaciones Científicas (IVIC). Departamento de Biología Estructural. Km 11 Carretera Panamericana. Altos de Pipe, Estado Miranda, Venezuela

## Abstract

**Background:**

The establishment of the cellular localization of proteins in *M. tuberculosis *will provide of valuable information for the identification of new drug/vaccine/diagnostic targets. Cytolocalization by inmunofluorescence microscopy has been limited in mycobacteria because to difficulties in effectively permeabilize it.

**Results:**

A treatment combining lysozyme with triton X-100 was found to be an effective permeabilization method of the mycobacterial envelope.

**Conclusion:**

A rapid and simple permeabilization protocol has been successfully assessed in pure cultures of both *Mycobacterium smegmatis *and *Mycobacterium tuberculosis *H37Rv. This method can be successful used in the cytolocalization of proteins by immunolabeling.

## Background

*Mycobacterium tuberculosis *is a facultative intracellular pathogen responsible for human tuberculosis (TB). TB is the second most common infectious cause of adult mortality and over 98% of deaths from TB occur in developing countries [14]. In the past few years there have been remarkable advances in the understanding of the biology of this pathogen mainly due to the completion of the genome sequence of the widely studied *M. tuberculosis *H37Rv strain [3], as well as the development of many tools and techniques that have taken advantage of the genome data. However, one field of proteomics that has been poorly exploited in mycobacteria is protein localization. Recent advances in microscopy and protein localization techniques have provided new insights into the remarkable complexity of the bacterial cell. Although, bacteria lack discrete cellular compartments such as organelles, many proteins are targeted to specific subcellular sites, which are essential for proper function and regulation. The information obtained by identifying these sites will be valuable in tracking targets suitable in drug/vaccine/diagnostic of *M. tuberculosis*. The few reports on cellular proteins localization in mycobacteria have used GFP -tagged proteins [4,7,11]. The main drawback to this technique is that no prediction can be made a priori as to whether a particular fusion construct will generate a functional and fluorescent protein, and in fact discrepancies in the localization and partial functionality of GFP-tagged proteins in bacteria has been reported [9]. An alternative method for subcellular localization is immune staining using antibodies against the protein in study. This technique has not been assayed in mycobacteria principally due to the difficulty in effectively permeabilize the cell envelope, a highly lipophilic barrier that protects the tubercle bacillus, comprising of mycolate residues and other lipids arranged as a closed-packed monolayer throughout the periphery of the bacteria [6]. Previous studies in mycobacteria have tested several protocols for fluorescence *in situ *hybridization (FISH); among them the hydrolysis with 1 M HCl and 80% (v/v) ethanol were found to be moderately effective [2,5,8,10]. However, we found that these treatments did not successfully permeate molecules such as antibodies into *M. smegmatis *or *M. tuberculosis *(data not shown).

In this work we describe a protocol to permeabilize the envelope of both *M. smegmatis *and *M. tuberculosis*.

## Results and discussion

We reasoned that the effective permeabilization of the mycobacterial envelope could be easily detected in cells bearing the pGFP22-4 plasmid, which constitutively expresses the GFP protein under the control of the *dnaA *gene promoter [12]. Due to the cytoplasmic nature of the GFP protein, mycobacteria bearing the pGFP22-4 plasmid are visualized as green bacilli in fluorescence microscopy. With successfully permeabilized cells, molecules such as antibodies can cross the cell envelope, the anti-GFP primary antibodies can interact with the intracellular GFP and the further hybridization with a Cy3-conjugated secondary antibody will enable the visualization of the mycobacteria as red bacilli.

**Figure 1:**
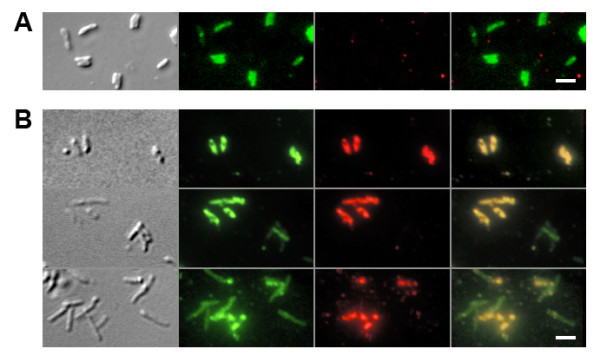
**Fluorescence microscopy of *M. tuberculosis *H37Rv (pGFP22-4) hybridized with anti-GFP and anti-mouse coupled to Cy3 as primary and secondary antibodies**. A) Without permeabilization treatment. B) After permeabilization with lysozyme (2 mg ml^-1^, 30 min, 37°C) and triton X-100 (0.1%, 5 min, RT). Left to right, DIC, Blue-2A, Cy3 and superposition of the fluorescent images. Bar = 3 μm.

The table I summarize the results obtained. No positive red fluorescence signal was obtained when the mycobacteria was treated only with triton X-100, independently of concentration (0.01% – 0.1%) and length of incubation (5, 10 and 20 min). In contrast, a strong red fluorescence signal was obtained when the bacilli were initially incubated for 30 min at 37°C with lysozyme (2 mg ml^-1^, prepared in water) followed by 5 min at RT with 0.1% triton X-100. Prolongation of the incubation time with triton X-100 for more than 5 min generated cellular lysis. No red fluorescent bacilli were observed when the lysozyme was dissolved in PBS. Figure 1 shows that under the conditions of permeabilization described above, the majority of the green fluorescent *M. tuberculosis *cells bearing the pGFP22-4 plasmid also showed red fluorescence, demonstrating that effectively the mycobacterial envelope was permeated. However, it was noted that when the initial cellular density was increased many green *M. tuberculosis *cells did not show red fluorescence, suggesting a decrease in the efficiency of permeabilization.

Following the methodology described in this work we have determined the subcellular localization of cell division proteins of *M. tuberculosis*, e.g. the Structural Maintenance of Chromosome (SMC) protein, as defined foci (Cimino and Salazar, unpublished).

## Conclusion

The data presented here clearly demonstrates that it is possible to successfully permeabilize mycobacteria with lysozyme and triton X-100, under the controlled conditions described in this work, as evidenced by its ability to allow bacterial uptake of specific antibodies. This work described a simple and efficient procedure to permeate the envelope of *M. tuberculosis *that can be successful used in the cytolocalization of proteins by immunolabeling.

## Methods

*M. smegmatis *mc^2^155 [13] and *M. tuberculosis *H37Rv (ATCC27294) bearing the pGFP22-4 plasmid were grown as described [12]. *M. tuberculosis *was grown and treated inside a BSL3 containment facility. Aliquots of 3 ml of the cultures were taken at early stationary growth phase, as determined by Colonies Forming Units (CFU) along the growth, harvested by centrifugation at 9000 × g for 5 min, washed three times in 1 × PBS (pH 7.4), resuspended in 1/3 – 1/6 volume of 4% paraformaldehyde (PFA) and incubated for 30 min at 37°C, followed by 50 mM ammonium chloride for 5 minutes at RT. In order to permeabilize the envelope of the mycobacteria, the cells were treated with triton X-100 alone or in combination with lysozyme under different concentrations, times and temperatures of incubation (Table I). Finally, the cells were washed twice in 1 × PBS and resuspended in 100–200 μl of PBS. Microscope slides were covered with 5 μl poly-L-lysine (Sigma) and excess poly-L-lysine was removed away with distillated water [1]. A 20 – 40 μl aliquot was applied to pre-treated slides, allowed to air dry and blocked with 2% BSA in 1 × PBS during 30 min at room temperature. An additional PFA fixation and treatment with ammonium chloride must be performed to avoid detachment of the cells. Incubation with the primary antibody, a mouse polyclonal antiserum to GFP (Clontech), was carried out for 30 min at room temperature at a dilution of 1:200 in 1 × PBS with 1% BSA. After three PBS rinses cells were incubated with a Cy3-conjugated secondary antibody to mouse IgG (1/100 dilution; Amersham), finally the cells were rinsed three times with PBS prior to microscopy. GFP and Cy3 fluorescence as well as Difference Interference Contrast (DIC) images were observed with a 100 X oil immersion (N.A. 1.3) objective on an Orca 12-ERG digital CCD camera (Hamamatsu Photonics) mounted on a Nikon E600 microscope fitted with Blue 2A (ex. 450–490 nm, em. 515 nm) and Cy3HQ (ex. 530 – 560 nm, em. 573 – 648 nm) filter cubes. Images were taken and processed by using Metamorph software (Universal Imaging Corporation).

## Authors' contributions

MC carried out the permeabilization experiments. LA performed the analysis and digitalization of the images. LS conceived of the study, participated in coordinating and supervising the study, and wrote the manuscript. All authors read and approved the final manuscript.

## Figures and Tables

**Table 1:** Permeabilization protocols

	Incubation time (min)	Temperature (°C)	Permeabilisation
Triton X-100 (0.01%)	5	RT	-
	10	RT	-
Triton X-100 (0.1%)	5	RT	-
	10	RT	-
	20	RT	-
Lysozyme (2 mg ml^-1 ^in H_2_O); Triton X-100 (0.1%, 5 min, RT)	30	RT	+
	30	37	++
	> 30	37	-^a^
Lysozyme (2 mg ml^-1 ^in PBS); Triton X-100 (0.1%, 5 min, RT)	30	37	-
Lysozyme (2 mg ml^-1 ^in H_2_O); Triton X-100 (0.1%, 10 min, RT);	30	37	-^a^

^a ^cellular lysis
